# Antidepressant Effects on Insulin Sensitivity and Proinflammatory Cytokines in the Depressed Males

**DOI:** 10.1155/2010/573594

**Published:** 2010-05-18

**Authors:** Yi-Chyan Chen, Wei-Win Lin, Yu-Jung Chen, Wei-Chung Mao, Yi-Jen Hung

**Affiliations:** ^1^Department of Psychiatry, Tri-Service General Hospital, National Defense Medical Center, Taipei 114, Taiwan; ^2^Department of Psychiatry, Hualien Armed Forces General Hospital, Hualien 97144, Taiwan; ^3^Division of Endocrinology and Metabolism, Tri-Service General Hospital, National Defense Medical Center, No. 325, Sec. 2, Cheng-Kung Rd, Taipei 114, Taiwan

## Abstract

Growing evidence suggests that mood disorder is associated with insulin resistance and inflammation. Thus the effects of antidepressants on insulin sensitivity and proinflammatory responses will be a crucial issue for depression treatment. In this study, we enrolled 43 non-diabetic young depressed males and adapted standard testing procedures to assess glucose metabolism during 4-week hospitalization. Before and after the 4-week antidepressant treatment, participants underwent oral glucose tolerance test (OGTT) and frequently sampled intravenous glucose tolerance test (FSIGT). Insulin sensitivity (S_I_), glucose effectiveness (S_G_), acute insulin response, and disposition index (DI) were estimated using the minimal model method. The plasma levels of C-reactive protein (CRP), interleukin-6 (IL-6), tumor necrosis factor-*α* (TNF-*α*), and adiponectin were measured. The Hamilton depression rating scale (HAM-D) total scores were reduced significantly during the course of treatment. There were no significant changes in the parameters of S_I_, S_G_, and DI. Compared to drug naïve status, the level of plasma IL-6 was significantly elevated (0.77 to 1.30 pg/ml; *P* = .001) after antidepressant therapy. However, the concentrations of CRP, TNF-*α*, and adiponectin showed no differences during the course of treatment. The results suggest that antidepressants may promote stimulatory effect on the IL-6 production in the early stage of antidepressant treatment.

## 1. Introduction

Depressive disorder is a complex and multifactorial disorder with biological heterogeneity. Growing evidence suggests the mood disorder is associated with insulin resistance and inflammation [1, 2]. Findings also suggest an overlapping pathophysiology between depressive disorder and insulin regulation. Some studies have demonstrated that depressed individuals had higher glucose levels and insulin resistance when they are symptomatic [3–6]. Epidemiologic evidences showed that obesity correlated with depression, and depressed adults have a 37% increased risk of developing type 2 diabetes [7–9]. Additionally, population-based surveys have also evidenced that the prevalence rate of depression in diabetes is around 8% to 25% and up to 40–80% for diabetic patients with physical complications [10–12]. Comorbid depression in diabetic patients could deter treatment adherence and increase the frequency of medical complications. Therefore, identifying depression and reversing negative mood have been cited as important issues for diabetes care [13, 14].

Most antidepressant medications, including tricyclic antidepressants (TCAs), selective serotonin reuptake inhibitors (SSRIs), serotonin norepinephrine reuptake inhibitors (SNRIs), and mirtazapine, increase levels of monoaminergic serotonin and norepinephrine, and modify the balance of the hypothalamus-pituitary-adrenal (HPA) axis known to be associated with depression as well as insulin resistance [15–17]. The cause-and-effect relationship between antidepressants and glucose metabolism is still unclear [18]. The noradrenergic nortriptyline used to treat depressive symptoms hinder glycemic control in diabetes patients [19]. Compared to TCAs, SSRI antidepressants were associated with an increased insulin sensitivity index and reduced glycosylated hemoglobin (HbA1c) in depressed patients [20–22]; however, a case study reported that subjects treated with SSRIs became hyperglycemic [23]. Weight gain related to mirtazapine has not been well established; however, Himmerich et al. reported that the weight gain is not parallel to glucose intolerance through a short treatment period [24]. Hence, the question of whether antidepressants influence the insulin resistance still remains unresolved.

Persistent depressed mood substantially increases the risk of cardiovascular disease via regulating the cellular immunoinflammatory responses. Formation of artherosclerosis represents a series of metabolic changes and cellular inflammatory responses [25]. Adipokines, such as tumor necrosis factor-*α*  (TNF-*α*), interleukin-6 (IL-6), and adiponectin secreted from adipocytes and immune cells are predictors of vascular disease and insulin resistance [26]. Several studies have reported that lower plasma adiponectin as well as higher levels of cytokines and C-reactive protein (CRP), coexist in type 2 diabetes and are contributory factors for developing cardiovascular disease [27]. Some evidences indicate that depressed mood could be accompanied by the activation of cellular inflammation, and antidepressants have modulatory effect on the production of cytokines, especially activation of IL-6 secretion [28–30]. 

 To date, there is no consensus on the relationship of antidepressants and glucose-insulin homeostasis in the early phase or long-term treatment regimens. In this study, the effects of antidepressants on insulin-glucose homeostasis and alterations of proinflammatory cytokines would be verified by a structured study procedure and homogenous depressed subjects.

## 2. Materials and Methods

### 2.1. Subjects and Study Design

Participants were between 20 and 25 years of age with a body mass index (BMI) less than 25 kg/m^2^. All patient participants were recruited from a single center to ensure patient homogeneity and uniform procedural conduct. Depressed subjects, who were Han Chinese and lived in Taiwan, were eligible for the study if their fasting plasma glucose was less than 6.1 mmol/L. No patients had a history of systemic disease or family history of diabetes. Persons using psychotropics or medications known to alter glucose metabolism were excluded from the study. Potential adverse events and study procedures were explained to all participants, and informed consent was obtained prior to study entry. This study was approved by the Institutional Review Board in TriService General Hospital for Human Studies.

 Forty-three depressed males, who met the *Diagnostic and Statistical Manual of Mental Disorder, fourth edition* (DSM-IV) diagnostic criteria of major depressive disorder with single episode, were recruited and interviewed by two psychiatrists. All of the subjects were drug free at least one week before participating in the study and were admitted to the psychiatric ward due to symptom disturbance and potential suicide risk. The severity of depression was evaluated with the 21-item Hamilton depression rating scale (HAM-D). Prior to drug randomization, the patients underwent an oral glucose tolerance test (OGTT) and frequently sampled intravenous glucose tolerance test (FSIGT) to ensure no remarkable metabolic problems at baseline. The participants were randomly assigned to one of four antidepressant treatment groups in a 1 : 1 : 1 : 1 manner, maprotiline (*n* = 11), fluoxetine (*n* = 11), venlafaxine (*n* = 10), and mirtazapine (*n* = 11), and titrated up to the optimal dosage range. 

 During hospitalization, a relatively uniform diet, was served as isocaloric (30 kcal/kg/day) meals derived from 15% protein, 33% fat, and 52% carbohydrate sources. Meals were contained 20%, 40%, and 40% of the daily total caloric intake in each meal, respectively Daily activity, and exercises were also provided. Except for study medications, other psychotropic drugs (except lorazepam) were not permitted. Alcohol and coffee were restricted during the study period. Four weeks following antidepressant treatment and psychiatric intervention, participants underwent repeated mental status evaluation, HAM-D rating, and OGTT and FSIGT examinations.

### 2.2. Oral Glucose Tolerance Test (OGTT)

After a 10-hour overnight fast, OGTT was performed at 8:30 am by orally administering a 75 g glucose load in 150 ml of free water. Venous blood samples were obtained for plasma glucose and insulin determination at 0, 30, 60, 90, 120, and 180 minutes after glucose ingestion. The glucose and insulin areas under curve in response to OGTT were determined. The indices of basal insulin resistance and *β*-cell function were assessed using the homeostasis model assessment (HOMA-IR and HOMA-*β*) originally described by Matthews et al. in which HOMA-IR (mmol/L ×  *μ*IU/mL) = fasting glucose (mmol/L) × fasting insulin (*μ*IU/mL)/22.5 and HOMA-*β* = fasting insulin (*μ*IU/mL) × 20/[fasting glucose (mmol/L) − 3.5] [31]. The insulinogenic index (measure of insulin secretion during the OGTT) was calculated as the total incremental plasma insulin divided by the total incremental plasma glucose during the 2-hour OGTT period (insulin area under the curve above the baseline/glucose area under the curve above the baseline; represented as ΔI/ΔG) [32].

### 2.3. Frequently Sampled Intravenous Glucose Tolerance Test (FSIGT)

An insulin-modified FSIGT with 12 blood samples was performed to evaluate insulin sensitivity. An intravenous 50% glucose solution bolus (0.3 g/kg) was injected smoothly within 60 seconds at time 0 and a bolus (0.025 U/kg) of regular human insulin (Novo Nordisk Pharmaceutical, Princeton, NJ, USA) was given 20 minutes later. Blood samples were collected at 0, 2, 4, 8, 19, 22, 30, 40, 50, 70, 100, and 180 minutes. 

 Whole-body insulin sensitivity (S_I_) was calculated using the Bergman minimal model [33]. Briefly, S_I_ is the increase in the fractional clearance rate of glucose per unit change in the plasma insulin concentration. Estimates of S_I_ from this model have been validated against the glucose clamp technique. Acute insulin response (AIR) is the increment in the plasma insulin concentration above baseline in the first 8 minutes after glucose administration and is used as a sensitive measure of *β*-cell function. S_G_ (glucose effectiveness, independent of insulin, on the glucose utilization rate) is the glucose concentration at time 0 estimated by extrapolating the prediction of the glucose kinetics model to the moment of injection and obtained by using a minimal model algorithm. The disposition index (DI) was defined as the product of AIR and S_I_ that measures the ability of pancreatic *β*-cell to compensate for insulin resistance [34].

### 2.4. Laboratory Measurements

Following a 10-hour fast, venous blood was obtained for determining plasma glucose, insulin, thyroid hormone, cortisol, blood urea nitrogen (BUN), creatinine, aspartate aminotransferase (AST), alanine aminotransferase (ALT), and lipid profiles. Plasma circulating adiponectin, CRP, TNF-*α*, and IL-6 levels were also measured. Biochemistry and serum total cholesterol were measured using a dry multilayer analytic slide method in the Fuji Dri-Chem 3000 analyzer (Fuji Photo Film Corporation, Minato-Ku, Tokyo, Japan). The determination of serum triglyceride after enzymatic splitting with lipoprotein lipase was assayed by colorimetric enzymatic test on Hitachi 717 (Biomedilines, San Diego, CA, USA), while the plasma glucose concentration was determined by the glucose oxidase method on a Beckman Glucose Analyzer II (Beckman Instruments, Fullerton, CA, USA).

 Plasma insulin was measured with a radioimmunoassay kit (Coat-A-Count Insulin Kit, Diagnostic Products Corporation, Los Angeles, CA, USA). The intra- and interassay coefficients of variance (CV) were 3.3% and 2.5%, respectively. Plasma CRP levels were measured using the Tina-quant (Latex) high sensitive assay (Roche Diagnostics GmbH, Mannheim, Germany). Cortisol assay was a competitive immunoassay using direct chemiluminescent technology (Bayer HealthCare LLC, Tarrytown, NY, USA). Thyroid stimulating hormone (TSH) was determined using Coat-A-Count TSH immunoradiometric assay (Los Angeles, CA, USA), while triiodothyronine (T3) and nonprotein-bound thyroxin (free T4) were assayed by the method of Coat-A-Count T3 and free T4 solid-phase ^125^I radioimmunoassay (Los Angeles, CA, USA). 

 Serum adiponectin concentrations were assayed with radioimmunoassay established by Linco Research (St Charles, MO, USA). Total adiponectin, in a range of high- to low-molecular weight multimeric forms, were included. This assay had a sensitivity of 1 ng/mL and intra- and interassay CV of less than 8%. Serum IL-6 concentrations were determined by the method of human high sensitivity Enzyme Linked-ImmunoSorbent Assay (ELISA) established by Diaclone Research (Besancon Cedex, France). The intra- and interassay CV for IL-6 were 1.4% and 5.5%, respectively. Serum TNF-*α* was measured with the Biotrak high sensitivity human ELISA kit from Amersham Biosciences (Buckinghamshire, UK). The minimal detectable dose of TNF-*α* was determined to be 0.1 pg/mL, by adding two standard deviations to the optical density value of zero and calculating the corresponding concentration from the standard curve. The intra- and interassay CV for TNF-*α* were 5.8% and 9.3%, respectively.

### 2.5. Statistical Analyses

The sample size determination was based on the power (*β*) in the response rate of depression. To determine whether the distribution of each variable approximates normal distribution in depressed group treated with different antidepressants, the Q-Q plot was adapted to test normality. Statistical differences in the demographic characteristics among the four groups of antidepressant treatment were calculated by one-way analysis of variance (ANOVA) with Tamhane's post hoc test. To compare the differences of glucose metabolic parameters, and adipokines before and after antidepressant treatment, paired *t* test was used. Statistical calculations were carried out using the SPSS program for Windows (11.5.0 version, SPSS, Inc., Chicago, IL, USA). All values were expressed as mean ± standard error (SEM).* P* values less than  .05 were considered statistically significant.

## 3. Results

The randomized treatment groups were similar for age, body weight, BMI and HAM-D scores. The baseline parameters of fasting glycemic index, liver, renal, thyroid functions and morning cortisol level were all within normal limits and similar between the treatment subgroups. Total cholesterol (159.6 ± 7.3 mg/dl) and total triglyceride (76.0 ± 5.4 mg/dl) were also within normal limits and similar between the treatment subgroups. 

 At the baseline, the HAM-D total score was 31.1 and decreased by 50% (*P* < .005) following inpatient treatment. Weight gain was found in maprotiline- and mirtazapine treated groups (1.3 and 1.6 kg, resp.). Although weight gain was observed in 2 of the 4 treatment groups during the course of intervention, the weight gain was not statistically significant and allowed the participants to be pooled for the remainder of analyses. 

 There were no statistical changes in the fasting and 2 hour-plasma glucose, and insulin concentrations, based on OGTT, after 4 weeks of antidepressant treatment. Furthermore, the values for HOMA-IR, HOMA-*β* and insulinogenic index remained unchanged following treatment (Table 1); moreover, no differences were found among the separated antidepressant subgroups by ANOVA testing.

**Figure 1:**
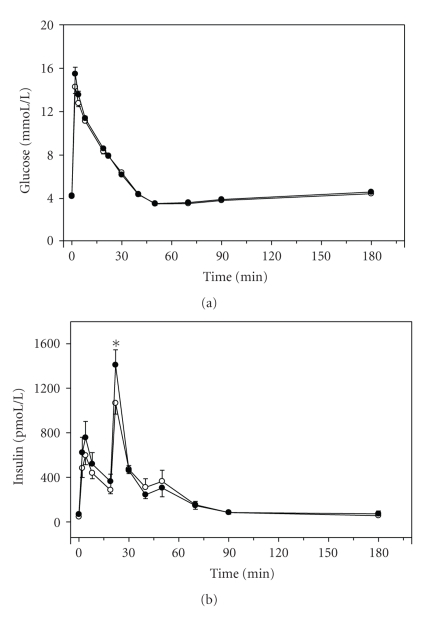
Blood concentrations of glucose (a) and insulin (b) following frequently sampled intravenous glucose tolerance test (FSIGT). The pre-treatment group (*N* = 43) is denoted as (∘), and posttreatment group is denoted as (●). Vertical bars (for clarity only the upper or lower portion is shown) represent standard error of the means.

**Figure 2:**
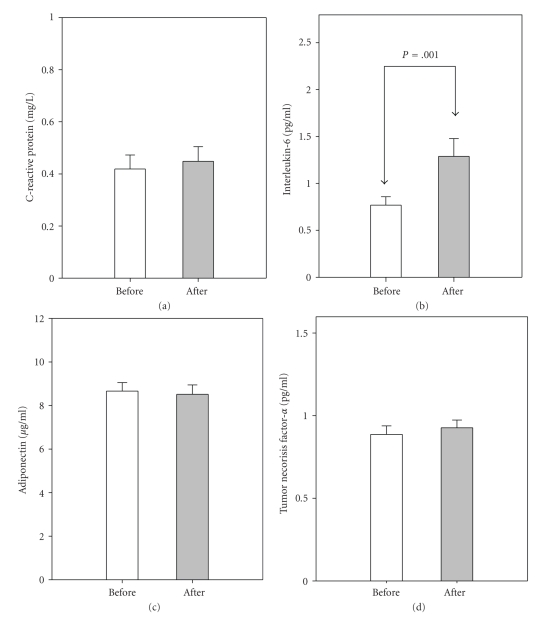
Alterations in plasma concentrations of CRP, cytokines and adiponectin in depressed males following antidepressant treatment. Open and gray bars represent as before and after antidepressant treatment, respectively.

The plasma levels of glucose and insulin from the FSIGT at baseline and posttreatment are presented in Figure 1. There were no statistical changes in plasma glucose at any time interval based on FSIGT between baseline and posttreatment. Elevation in plasma insulin at 22-minutes post glucose injection was significantly higher posttreatment than observed at baseline. To determine insulin sensitivity and pancreatic B-cell function, we used Bergman's minimal model. No significant changes in insulin sensitivity or B-cell function were observed (Table 1), even analyzed in separated antidepressant groups. Plasma IL-6 was significantly elevated following the 4 week inpatient pharmaceutical intervention (Figure 2). However, CRP, TNF-*α*, and adiponectin remained unchanged.

## 4. Discussion

This is the first study demonstrating that short-term anti-depressant treatment is associated with reduced depressive symptoms and minimal anthropometric and metabolic changes in a homogenous group of young Asian men. In the study design, we recruited the nonobese subjects with normal BMI (22.6 ± 0.3 kg/m^2^) and the participants did not show metabolic impairment at the baseline. In the short-term antidepressant treatment, the overall response rate was approximately up to 50%. There was weight gain in the maprotiline and mirtazapine groups, but did not show significant difference. The finding of maprotiline and mirtazapine-related weight gain is consistent with previous literature reports; however, there was no raising body weight by fluoxetine and venlafaxine treatment in this study. Comparing to previous literature, the SSRIs or SNRIs-related weight change is still inconclusive issues [15, 35]. 

Our findings showed no significant differences in insulin resistance or offsetting beta-cell secretion defined by OGTT and FSIGT following antidepressant treatment. Regarding the relationships between antidepressants and insulin sensitivity, there are some discrepancies between our findings and the previous studies [18, 21, 23, 36–39]. In preclinical animal studies, Erenmenisoglu et al. investigated the metabolic difference between TCA and SSRI in mice and found that TCA treatment induced hyperglycemia and hyperinsulinemia; in contrast, the SSRI-treated group had reduced blood glucose levels [40]. This finding has been refuted by Yamada et al. and Gomez et al. found that SSRIs could induce hyperglycemia and increase plasma insulin levels in male rats and mice models [39, 41]. From the clinical survey, TCA's medications could induce weight gain and have adverse effects in glycemic control for diabetes patients [19]; however, SSRI antidepressants were able to decrease the fasting glucose level and reduce level of glycosylated hemoglobin in depressed patients [20–22]. To clarify these controversial issues, Okamura et al. used FSIGT procedure and minimal model analysis and demonstrated insulin sensitivity in depressed patients treated with tri/tetracyclic antidepressants showed improvement through 3-month treatment [35]. Compared to Himmerich's results, it demonstrated that the glucose tolerance in the OGTT was not correlated with mirtazapine-induced weight gain [24]. Reviewing the literatures, we propose the plausible discrepancy may be due to sampling variations such as body weight, age, diet and concomitant psychotropic medications. To control these sampling diversities in this study, we recruited inpatient young depressed males, adjusted the diet and conducted the structured procedures during the course of treatment. Thus, this study would have the strength to clarify the relationship between antidepressants and insulin resistance. 

Adiponectin is an adipocyte-derived hormone that is inversely associated with insulin sensitivity and body weight. In Figure 2, is resulted demonstrates without difference of adiponectin level following antidepressant treatment, it would be probably attributed to insufficient change of insulin sensitivity and weight gain in this study. Growing evidence shows elevation of plasma IL-6 and TNF-*α* concentrations in depressed and diabetic patients and IL-6 represents the higher association with obesity and insulin resistance [42, 43]. TNF-*α* is an important stimulator of a second wave of cytokines, including IL-6 and other chemokines. Hinze-Selch et al. demonstrated that the activation of TNF-*α* and weight gain was induced by TCA and paroxetine antidepressants during the course of treatment [44]. IL-6 is also secreted in response to stress via enhancing noradrenergic neurotransmission and stimulating the HPA axis which are regulated by antidepressants [25]. Interestingly, our result demonstrated the plasma IL-6 was significantly elevated and independent of weight change, adiponectin and TNF-*α* production during short-term antidepressant treatment; besides, not parallel to the alteration of TNF-*α*. The significant elevation of IL-6 may be attributed to the antidepressant modulatory effects via noradrenergic and/or serotonergic transmission on the adipose tissues and immune systems in the early antidepressant-treated course. However, whether the increasing level of IL-6 is directly responsible antidepressants themselves is still an undefined issue. Furthermore, the balancing effect between the inflammatory IL-6 cytokine and anti-inflammatory cytokines (e.g., IL-10) would need to clarified in the future. 

 There were some limitations and weakness in this study. First, it was lack of the corresponding controls, such as untreated depressed group or nondepressed with antidepressant-treated cohort. Second, the unequal and limited sample size in the different subgroups of antidepressants may restrict the statistical power and have potential chance of type I error statistically. It is an intriguing issue to explore the effects of the different antidepressants on glucose metabolism, and the different pharmacological mechanisms and diverse neurotransmissions can influence the insulin secretion and proinflammatory cytokine release. 

 In summary, our study provides a homogenous condition to clarify the glucose-insulin homeostasis in relation to depression and antidepressant treatment. The results suggest that antidepressants may lead to stimulatory effect on the IL-6 production in the early stage of antidepressant treatment; however, the separated antidepressant and long-term effects still need to be clarified by using larger samples.

## Figures and Tables

**Table 1:** Differences of body weight, depressive symptoms, and metabolic parameters of glucose metabolism in depressed patients before and after treatment.

	Before	After
	(*N* = 43, mean ± SE)	(*N* = 43, mean ± SE)
Age (years)	23.3 ± 0.4	22.3 ± 0.4
Body weight (kg)	65.7 ± 1.0	65.8 ± 1.1
BMI (kg/m^2^)	22.6 ± 0.3	22.1 ± 0.3
Systolic BP (mmHg)	119.6 ± 1.6	117.9 ± 1.4
Diastolic BP (mmHg)	76.8 ± 1.2	75.3 ± 0.9
HAM-D	31.1 ± 1.5	16.5 ± 1.7**
HOMA-IR	1.11 ± 0.14	1.25 ± 0.15
HOMA-*β*	154.74 ± 56.20	273.09 ± 92.30
ΔI/ΔG (*μ*IU/mg)	144.2 ± 13.8	149.9 ± 17.4
S_I_ (10^−5^ min^−1^/pmol)	0.90 ± 0.09	0.70 ± 0.08
S_G_ (min^−1^)	0.028 ± 0.003	0.035 ± 0.007
AIR (pmol)	3440 ± 424	4100 ± 419
DI (S_I_ × AIR)	2871 ± 400	2968 ± 407

*P < .05

**P < .005.

## Abbreviations

BMI: Body mass index
BP: Blood pressure
HAM-D: 21 items of Hamilton depression rating scale
HOMA-IR: Homeostasis model of assessment for insulin resistance
HOMA-*β*: Homeostasis model of assessment for pancreatic *β*-cell secretory function
ΔI/ΔG: Insulinogenic index (insulin production during the OGTT)
S_I_: Insulin sensitivity
S_G_: Glucose effectiveness
AIR: Acute insulin response to intravenous glucose load
DI: Disposition index.
